# 1-(2-Hy­droxy­eth­yl)-3-[(2-hy­droxy­eth­yl)amino]-4-(1*H*-indol-3-yl)-1*H*-pyrrole-2,5-dione

**DOI:** 10.1107/S1600536811007677

**Published:** 2011-03-12

**Authors:** Zhi-Xiong Xie, Sheng-Yin Zhao

**Affiliations:** a College of Chemisty, Chemical Engineering and Biotechnology, Donghua University, Shanghai 201620, People’s Republic of China

## Abstract

There are four molecules in the asymmetric unit of the title compound, C_16_H_17_N_3_O_4_, in which the dihedral angles between the indole ring system and maleimide ring are 4.5 (3), 8.3 (3), 8.4 (2) and 10.4 (2)°. In the crystal, mol­ecules are linked by numerous N—H⋯O and O—H⋯O hydrogen bonds, generating a three-dimensional network.

## Related literature

For general background to indolylmaleimides and their biological properties, see: Vegesna *et al.* (1998) ▶; Hu (1996 ▶); Zhao *et al.* (2008 ▶). For the preparation, see: Zhao *et al.* (2010 ▶).
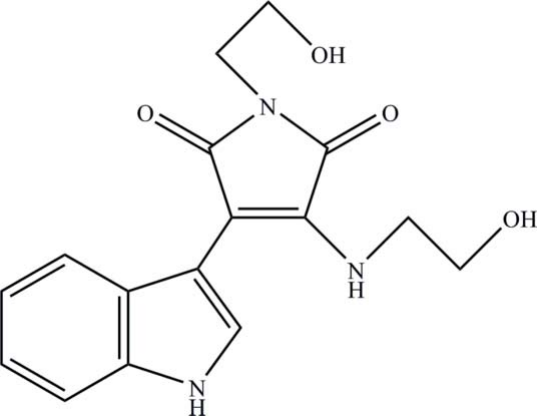


## Experimental

### 

#### Crystal data

C_16_H_17_N_3_O_4_
*M* *_r_* = 315.33
Triclinic, 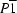

*a* = 13.0276 (10) Å
*b* = 14.8150 (11) Å
*c* = 17.8893 (14) Å
α = 90.012 (1)°
β = 110.295 (2)°
γ = 102.187 (2)°
*V* = 3155.2 (4) Å^3^
*Z* = 8
Mo *K*α radiation
μ = 0.10 mm^−1^
*T* = 293 K
0.43 × 0.33 × 0.27 mm

#### Data collection

Bruker SMART CCD diffractometer
Absorption correction: multi-scan (*SADABS*; Bruker, 2003 ▶) *T*_min_ = 0.594, *T*_max_ = 1.000
17464 measured reflections
12193 independent reflections
6708 reflections with *I* > 2σ(*I*)
*R*_int_ = 0.035

#### Refinement

*R*[*F* ^2^ > 2σ(*F* ^2^)] = 0.047
*wR*(*F* ^2^) = 0.098
*S* = 0.89
12193 reflections
893 parameters
13 restraints
H atoms treated by a mixture of independent and constrained refinement
Δρ_max_ = 0.18 e Å^−3^
Δρ_min_ = −0.18 e Å^−3^

### 

Data collection: *SMART* (Bruker, 2003 ▶); cell refinement: *SAINT* (Bruker, 2003 ▶); data reduction: *SAINT*; program(s) used to solve structure: *SHELXS97* (Sheldrick, 2008 ▶); program(s) used to refine structure: *SHELXL97* (Sheldrick, 2008 ▶); molecular graphics: *SHELXTL* (Sheldrick, 2008 ▶); software used to prepare material for publication: *SHELXTL*.

## Supplementary Material

Crystal structure: contains datablocks I, global. DOI: 10.1107/S1600536811007677/hb5807sup1.cif

Structure factors: contains datablocks I. DOI: 10.1107/S1600536811007677/hb5807Isup2.hkl

Additional supplementary materials:  crystallographic information; 3D view; checkCIF report

## Figures and Tables

**Table 1:** Hydrogen-bond geometry (Å, °)

*D*—H⋯*A*	*D*—H	H⋯*A*	*D*⋯*A*	*D*—H⋯*A*
N2—H2⋯O12^i^	0.86 (3)	2.09 (3)	2.950 (3)	177 (3)
O3—H3⋯O8^ii^	0.84 (3)	1.93 (3)	2.762 (3)	172 (3)
O4—H4⋯O14^iii^	0.82 (2)	1.97 (2)	2.770 (2)	163 (3)
N5—H5⋯O16^iv^	0.83 (2)	2.22 (2)	3.027 (3)	164 (2)
O7—H7⋯O4^v^	0.86 (2)	1.89 (2)	2.755 (3)	175 (3)
O15—H15⋯O1^vi^	0.84 (2)	2.22 (2)	2.893 (2)	137 (3)
O15—H15⋯O12^vii^	0.84 (2)	2.42 (2)	3.097 (3)	139 (3)
O16—H16⋯O11^i^	0.83 (2)	1.96 (2)	2.780 (3)	170 (3)
N3—H3*A*⋯O6^iii^	0.87 (2)	2.06 (2)	2.840 (2)	149 (2)
N6—H6*A*⋯O14	0.84 (2)	2.22 (2)	3.042 (3)	168 (2)
O8—H8*A*⋯O10^viii^	0.85 (2)	1.90 (2)	2.746 (3)	173 (3)
N9—H9*A*⋯O2^viii^	0.89 (2)	1.95 (2)	2.760 (2)	152 (2)
O11—H11*A*⋯O5^ii^	0.87 (2)	2.13 (2)	2.935 (2)	154 (3)
O11—H11*A*⋯O16	0.87 (2)	2.51 (3)	3.033 (3)	120 (3)
O12—H12*A*⋯O15^ix^	0.84 (2)	1.92 (2)	2.739 (2)	168 (3)
N8—H8*B*⋯O7^x^	0.89 (2)	2.09 (2)	2.867 (3)	146 (2)
N11—H11*B*⋯O3	0.88 (2)	2.17 (2)	2.945 (3)	147 (2)
N12—H12*B*⋯O10^viii^	0.85 (2)	2.10 (2)	2.925 (2)	164 (2)

Symmetry codes: (i) 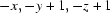; (ii) 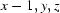; (iii) 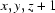; (iv) 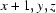; (v) 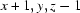; (vi) 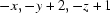; (vii) 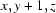; (viii) 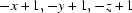; (ix) 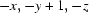; (x) 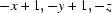.

**Fig. 1.**
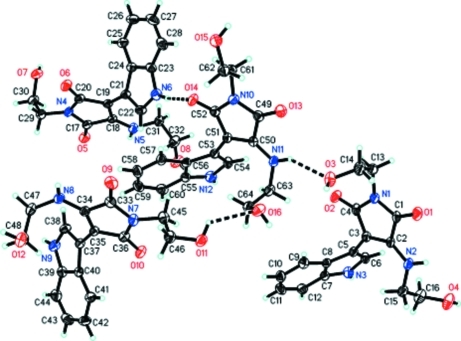
The molecular structure of (I) showing displacement ellipsoids drawn at the 30% probability level. H atoms are represented as small spheres of arbitrary radii. Hydrogen bonds are shown as dashed lines.

**Fig. 2.**
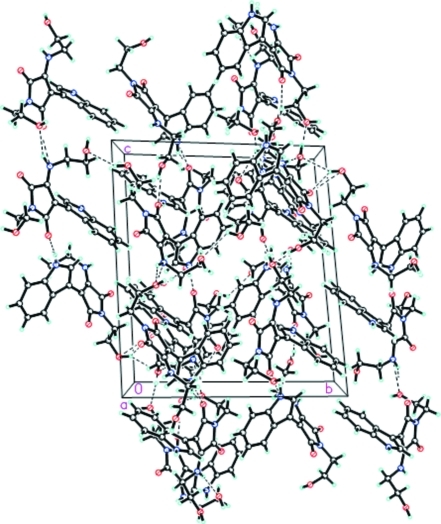
Packing of the title compound with hydrogen bonding shown as dashed lines.

## supplementary crystallographic information

### Comment

The indolylmaleimides and their derivatives derived from staurosporine, is a specific class of protein kinase C inhibitors,and can be used to treat or prevent a variety of conditions and diseases,such as an inflammatory diseasea or some kinds of cancer disease and so on (Vegesna *et al.*, 1998; Hu, 1996; Zhao *et al.*, 2008). The pharmacological results showed that the title compound displayed potent cytotoxic activity. Our reserach group has focused on the synthesis of the indolylmaleimides and their derivatives and we now report the structure of the title compound, (I).

The X-ray structural analysis confirmed the assignment of its structure from spectroscopic data. The asymmetric unit of (I) contains four independent molecules, each molecules are linked to each other through N—H···O and O—H···O intermolecular hydrogen bonds(Table 1, Fig. 1).The four molecules show slightly different conformations: the dihedral angles between the indole ring and maleimide ring are 4.5 (3), 8.4 (2),10.4 (2) and 8.3 (3)° in the four independent molecules.

In the crystal (Fig. 2),indolylmaleimide molecules are linked by intermolecular N—H···O hydrogen bonds. The layers are further connected into a three-dimensional network by N—H···O and O—H···O hydrogen bonds involving the maleimide molecules as H-donors and by weak π–π stacking interactions involving neighbouring indole and maleimide rings.

### Experimental

3-Bromo-4-(1*H*-indol-3-yl)-1*H*-pyrrole-2,5-dione (0.5 g, 1.67 mmol), 2-aminoethanol(0.15 g, 2.46 mmol) and 1.5 ml triethylamine was added to DMF (8 ml) in a flask. The reaction mixture was stirred at 363 K under argon for 10 h. After the completion of the reaction (determined by TLC), the reaction mixture was cooled to room temperature, and diluted with ethylacetate (80 ml). The solution was washed successively with water (50 ml), brine (50 ml) and then dried with anhydrous magnesium sulfate. The crude material was purified by chromatography with 2:1(hexane-EtOAc) as red crystals (0.31 g, 61%). The crystals were obtained by dissolving the title compound (0.3 g, 1.0 mmol) in ethyl acetate (5 ml) and ethanol (5 ml) and evaporating the solvent slowly at room temperature for about 2 d to yield red blocks.

### Refinement

The H atoms were placed at idealised positions and refined as riding (C—H in the range 0.93 – 0.97 Å) and *U*_iso_(H) (in the range 1.2 - 1.5 *U*_eq_(parent atom)]

### Crystal data

**Table:**

C_16_H_17_N_3_O_4_	*Z* = 8
*M**_r_* = 315.33	*F*(000) = 1328
Triclinic, *P*1	*D*_x_ = 1.328 Mg m^−^^3^
Hall symbol: -P 1	Mo *K*α radiation, λ = 0.71073 Å
*a* = 13.0276 (10) Å	Cell parameters from 3141 reflections
*b* = 14.8150 (11) Å	θ = 4.9–45.4°
*c* = 17.8893 (14) Å	µ = 0.10 mm^−^^1^
α = 90.012 (1)°	*T* = 293 K
β = 110.295 (2)°	Prism, red
γ = 102.187 (2)°	0.43 × 0.33 × 0.27 mm
*V* = 3155.2 (4) Å^3^	

### Data collection

**Table:**

Bruker SMART CCD diffractometer	12193 independent reflections
Radiation source: fine-focus sealed tube	6708 reflections with *I* > 2σ(*I*)
graphite	*R*_int_ = 0.035
ω scans	θ_max_ = 26.0°, θ_min_ = 1.2°
Absorption correction: multi-scan (*SADABS*; Bruker, 2003)	*h* = −16→8
*T*_min_ = 0.594, *T*_max_ = 1.000	*k* = −18→18
17464 measured reflections	*l* = −22→22

### Refinement

**Table:**

Refinement on *F*^2^	Primary atom site location: structure-invariant direct methods
Least-squares matrix: full	Secondary atom site location: difference Fourier map
*R*[*F*^2^ > 2σ(*F*^2^)] = 0.047	Hydrogen site location: inferred from neighbouring sites
*wR*(*F*^2^) = 0.098	H atoms treated by a mixture of independent and constrained refinement
*S* = 0.89	*w* = 1/[σ^2^(*F*_o_^2^) + (0.0246*P*)^2^] where *P* = (*F*_o_^2^ + 2*F*_c_^2^)/3
12193 reflections	(Δ/σ)_max_ = 0.002
893 parameters	Δρ_max_ = 0.18 e Å^−^^3^
13 restraints	Δρ_min_ = −0.18 e Å^−^^3^

### Special details

**Table:**

Geometry. All e.s.d.'s (except the e.s.d. in the dihedral angle between two l.s. planes) are estimated using the full covariance matrix. The cell e.s.d.'s are taken into account individually in the estimation of e.s.d.'s in distances, angles and torsion angles; correlations between e.s.d.'s in cell parameters are only used when they are defined by crystal symmetry. An approximate (isotropic) treatment of cell e.s.d.'s is used for estimating e.s.d.'s involving l.s. planes.
Refinement. Refinement of *F*^2^ against ALL reflections. The weighted *R*-factor *wR* and goodness of fit *S* are based on *F*^2^, conventional *R*-factors *R* are based on *F*, with *F* set to zero for negative *F*^2^. The threshold expression of *F*^2^ > σ(*F*^2^) is used only for calculating *R*-factors(gt) *etc*. and is not relevant to the choice of reflections for refinement. *R*-factors based on *F*^2^ are statistically about twice as large as those based on *F*, and *R*- factors based on ALL data will be even larger.

### Fractional atomic coordinates and isotropic or equivalent isotropic displacement parameters (Å^2^)

**Table:**

	*x*	*y*	*z*	*U*_iso_*/*U*_eq_	
O1	0.01888 (14)	0.90744 (12)	0.80210 (9)	0.0565 (5)	
O2	0.29635 (13)	0.85115 (12)	0.72416 (9)	0.0504 (5)	
O3	0.04854 (17)	0.83153 (15)	0.57712 (11)	0.0565 (6)	
O4	0.15970 (17)	0.77882 (16)	1.08999 (12)	0.0692 (6)	
O5	0.95648 (14)	0.59360 (11)	0.28315 (9)	0.0535 (5)	
O6	0.67689 (14)	0.66239 (12)	0.07237 (9)	0.0513 (5)	
O7	0.94607 (17)	0.67135 (14)	0.05474 (11)	0.0538 (5)	
O8	0.84197 (16)	0.71019 (15)	0.51014 (12)	0.0638 (6)	
O9	0.07500 (14)	0.42150 (11)	0.14849 (9)	0.0545 (5)	
O10	0.27077 (13)	0.32299 (11)	0.38483 (9)	0.0452 (4)	
O11	−0.03045 (15)	0.43298 (14)	0.37925 (10)	0.0511 (5)	
O12	0.03693 (16)	0.11914 (13)	0.04475 (11)	0.0539 (5)	
O13	0.07026 (14)	0.90731 (11)	0.37576 (10)	0.0518 (5)	
O14	0.28453 (13)	0.81144 (11)	0.25196 (9)	0.0451 (4)	
O15	−0.03425 (16)	0.92783 (14)	0.10409 (10)	0.0547 (5)	
O16	0.03196 (16)	0.62001 (13)	0.47197 (11)	0.0551 (5)	
N1	0.14842 (15)	0.89266 (13)	0.74548 (10)	0.0372 (5)	
N2	0.11889 (18)	0.78733 (15)	0.91611 (12)	0.0465 (6)	
N3	0.47775 (18)	0.70845 (14)	0.96808 (12)	0.0434 (6)	
N4	0.82676 (16)	0.61586 (13)	0.16343 (11)	0.0388 (5)	
N5	0.86558 (18)	0.71354 (15)	0.35412 (12)	0.0455 (6)	
N6	0.50460 (18)	0.79428 (15)	0.23571 (13)	0.0466 (6)	
N7	0.15305 (15)	0.37924 (13)	0.27663 (11)	0.0392 (5)	
N8	0.19072 (18)	0.29920 (14)	0.10367 (12)	0.0425 (5)	
N9	0.50170 (18)	0.18774 (15)	0.26645 (13)	0.0460 (6)	
N10	0.15776 (15)	0.86570 (13)	0.29283 (11)	0.0385 (5)	
N11	0.19009 (17)	0.79605 (14)	0.48792 (12)	0.0422 (5)	
N12	0.51134 (18)	0.68804 (15)	0.49777 (13)	0.0451 (6)	
C1	0.0978 (2)	0.87718 (16)	0.80078 (13)	0.0370 (6)	
C2	0.15798 (19)	0.81481 (15)	0.85866 (13)	0.0333 (6)	
C3	0.24264 (18)	0.79819 (15)	0.83623 (13)	0.0330 (6)	
C4	0.23771 (19)	0.84653 (16)	0.76469 (14)	0.0371 (6)	
C5	0.33199 (18)	0.74941 (15)	0.87521 (13)	0.0325 (6)	
C6	0.41135 (19)	0.77124 (16)	0.95001 (13)	0.0396 (6)	
H6	0.4191	0.8218	0.9839	0.048*	
C7	0.44387 (19)	0.64506 (16)	0.90381 (14)	0.0370 (6)	
C8	0.35307 (18)	0.66880 (15)	0.84379 (13)	0.0313 (5)	
C9	0.30427 (19)	0.61489 (16)	0.77103 (14)	0.0411 (6)	
H9	0.2443	0.6291	0.7300	0.049*	
C10	0.3471 (2)	0.54000 (16)	0.76140 (16)	0.0496 (7)	
H10	0.3161	0.5039	0.7130	0.060*	
C11	0.4362 (2)	0.51745 (17)	0.82295 (17)	0.0538 (7)	
H11	0.4623	0.4658	0.8149	0.065*	
C12	0.4857 (2)	0.56879 (17)	0.89430 (15)	0.0470 (7)	
H12	0.5452	0.5535	0.9350	0.056*	
C13	0.1222 (2)	0.95368 (16)	0.68137 (13)	0.0449 (7)	
H13A	0.1871	0.9734	0.6656	0.054*	
H13B	0.1066	1.0085	0.7009	0.054*	
C14	0.0232 (2)	0.90832 (18)	0.60956 (14)	0.0564 (8)	
H14A	−0.0420	0.8873	0.6248	0.068*	
H14B	0.0060	0.9526	0.5697	0.068*	
C15	0.1534 (2)	0.71408 (16)	0.96736 (13)	0.0446 (7)	
H15A	0.1938	0.6811	0.9445	0.054*	
H15B	0.0867	0.6703	0.9677	0.054*	
C16	0.2259 (2)	0.7482 (2)	1.05170 (14)	0.0603 (8)	
H16A	0.2576	0.6988	1.0796	0.072*	
H16B	0.2872	0.7989	1.0524	0.072*	
C17	0.8788 (2)	0.62661 (16)	0.24387 (14)	0.0383 (6)	
C18	0.82312 (19)	0.68873 (15)	0.27522 (13)	0.0338 (6)	
C19	0.73717 (18)	0.70832 (15)	0.21321 (13)	0.0327 (6)	
C20	0.73834 (19)	0.66356 (16)	0.14114 (14)	0.0376 (6)	
C21	0.64887 (18)	0.75675 (15)	0.21160 (13)	0.0340 (6)	
C22	0.57272 (19)	0.73278 (16)	0.24882 (13)	0.0411 (6)	
H22	0.5679	0.6819	0.2788	0.049*	
C23	0.5339 (2)	0.85913 (16)	0.18788 (14)	0.0399 (6)	
C24	0.62425 (19)	0.83761 (16)	0.17098 (13)	0.0353 (6)	
C25	0.6702 (2)	0.89384 (16)	0.12269 (14)	0.0448 (7)	
H25	0.7300	0.8812	0.1108	0.054*	
C26	0.6254 (2)	0.96828 (17)	0.09301 (15)	0.0578 (8)	
H26	0.6545	1.0057	0.0600	0.069*	
C27	0.5366 (3)	0.98819 (18)	0.11196 (17)	0.0637 (9)	
H27	0.5088	1.0395	0.0918	0.076*	
C28	0.4897 (2)	0.93519 (18)	0.15875 (15)	0.0539 (8)	
H28	0.4306	0.9491	0.1708	0.065*	
C29	0.8468 (2)	0.55311 (17)	0.11019 (14)	0.0490 (7)	
H29A	0.7846	0.5426	0.0595	0.059*	
H29B	0.8495	0.4940	0.1332	0.059*	
C30	0.9545 (2)	0.58963 (18)	0.09541 (15)	0.0553 (8)	
H30A	1.0171	0.6031	0.1459	0.066*	
H30B	0.9676	0.5435	0.0634	0.066*	
C31	0.83718 (19)	0.78606 (16)	0.39233 (13)	0.0440 (7)	
H31A	0.7932	0.8196	0.3514	0.053*	
H31B	0.9058	0.8294	0.4243	0.053*	
C32	0.7726 (2)	0.75013 (19)	0.44452 (15)	0.0561 (8)	
H32A	0.7490	0.8003	0.4642	0.067*	
H32B	0.7060	0.7038	0.4141	0.067*	
C33	0.13398 (19)	0.37915 (16)	0.19553 (14)	0.0392 (6)	
C34	0.20150 (18)	0.31586 (15)	0.17965 (13)	0.0335 (6)	
C35	0.26342 (18)	0.28810 (15)	0.25086 (12)	0.0310 (5)	
C36	0.23384 (18)	0.32799 (16)	0.31208 (14)	0.0356 (6)	
C37	0.35094 (18)	0.23509 (15)	0.26947 (13)	0.0321 (6)	
C38	0.43740 (19)	0.25097 (16)	0.24109 (13)	0.0395 (6)	
H38	0.4505	0.2982	0.2090	0.047*	
C39	0.4593 (2)	0.13065 (16)	0.31364 (14)	0.0396 (6)	
C40	0.36477 (18)	0.15878 (15)	0.31827 (12)	0.0317 (6)	
C41	0.3088 (2)	0.11198 (16)	0.36590 (13)	0.0415 (6)	
H41	0.2459	0.1288	0.3699	0.050*	
C42	0.3489 (2)	0.04011 (17)	0.40695 (14)	0.0536 (7)	
H42	0.3140	0.0096	0.4402	0.064*	
C43	0.4413 (2)	0.01276 (18)	0.39907 (16)	0.0573 (8)	
H43	0.4652	−0.0368	0.4262	0.069*	
C44	0.4973 (2)	0.05634 (18)	0.35303 (15)	0.0521 (7)	
H44	0.5584	0.0374	0.3480	0.062*	
C45	0.11261 (18)	0.43681 (16)	0.32088 (14)	0.0424 (6)	
H45A	0.1077	0.4950	0.2965	0.051*	
H45B	0.1655	0.4505	0.3754	0.051*	
C46	−0.00130 (19)	0.38894 (16)	0.32173 (14)	0.0451 (7)	
H46A	−0.0574	0.3888	0.2692	0.054*	
H46B	−0.0011	0.3250	0.3334	0.054*	
C47	0.2206 (2)	0.22208 (17)	0.07274 (14)	0.0439 (7)	
H47A	0.3002	0.2254	0.0996	0.053*	
H47B	0.2067	0.2268	0.0161	0.053*	
C48	0.1548 (2)	0.13003 (17)	0.08450 (14)	0.0489 (7)	
H48A	0.1791	0.0807	0.0644	0.059*	
H48B	0.1705	0.1246	0.1412	0.059*	
C49	0.1340 (2)	0.86713 (16)	0.36197 (14)	0.0383 (6)	
C50	0.20470 (18)	0.80888 (15)	0.41787 (13)	0.0332 (6)	
C51	0.27068 (18)	0.78090 (15)	0.38135 (13)	0.0313 (6)	
C52	0.24286 (19)	0.81751 (15)	0.30282 (13)	0.0354 (6)	
C53	0.36095 (18)	0.73141 (15)	0.41251 (13)	0.0314 (5)	
C54	0.44338 (19)	0.74950 (16)	0.48690 (13)	0.0399 (6)	
H54	0.4518	0.7966	0.5244	0.048*	
C55	0.47595 (19)	0.62988 (16)	0.42986 (14)	0.0382 (6)	
C56	0.38155 (18)	0.65533 (15)	0.37388 (13)	0.0336 (6)	
C57	0.3320 (2)	0.60708 (16)	0.29817 (14)	0.0419 (6)	
H57	0.2707	0.6231	0.2598	0.050*	
C58	0.3747 (2)	0.53610 (17)	0.28139 (16)	0.0519 (7)	
H58	0.3423	0.5043	0.2310	0.062*	
C59	0.4657 (2)	0.51040 (17)	0.33818 (19)	0.0589 (8)	
H59	0.4920	0.4611	0.3252	0.071*	
C60	0.5177 (2)	0.55631 (17)	0.41323 (17)	0.0526 (7)	
H60	0.5782	0.5388	0.4512	0.063*	
C61	0.11773 (19)	0.92236 (16)	0.22703 (13)	0.0419 (6)	
H61A	0.1654	0.9280	0.1952	0.050*	
H61B	0.1229	0.9840	0.2484	0.050*	
C62	−0.00267 (19)	0.88101 (16)	0.17422 (13)	0.0458 (7)	
H62A	−0.0107	0.8161	0.1600	0.055*	
H62B	−0.0521	0.8851	0.2034	0.055*	
C63	0.2175 (2)	0.71998 (17)	0.53710 (13)	0.0447 (7)	
H63A	0.2026	0.7276	0.5860	0.054*	
H63B	0.2968	0.7214	0.5516	0.054*	
C64	0.1493 (2)	0.62799 (17)	0.49328 (14)	0.0490 (7)	
H64A	0.1660	0.6200	0.4452	0.059*	
H64B	0.1712	0.5788	0.5268	0.059*	
H2	0.076 (2)	0.8158 (18)	0.9294 (16)	0.090 (11)*	
H3	−0.011 (3)	0.790 (2)	0.5591 (19)	0.110 (15)*	
H4	0.197 (2)	0.800 (2)	1.1362 (12)	0.115 (14)*	
H5	0.9167 (16)	0.6879 (14)	0.3791 (12)	0.049 (8)*	
H7	1.0115 (17)	0.7078 (19)	0.0656 (18)	0.108 (14)*	
H15	−0.028 (2)	0.9853 (12)	0.1074 (17)	0.091 (12)*	
H16	0.023 (2)	0.602 (2)	0.5139 (13)	0.101 (13)*	
H3A	0.5346 (18)	0.7095 (15)	1.0115 (13)	0.052 (8)*	
H6A	0.4477 (16)	0.7952 (16)	0.2469 (14)	0.058 (9)*	
H8A	0.811 (3)	0.698 (2)	0.5447 (16)	0.135 (16)*	
H9A	0.5621 (17)	0.1857 (18)	0.2545 (15)	0.080 (10)*	
H11A	−0.033 (3)	0.4887 (15)	0.366 (2)	0.154 (17)*	
H12A	0.027 (2)	0.107 (2)	−0.0032 (11)	0.093 (12)*	
H8B	0.1419 (16)	0.3246 (14)	0.0672 (11)	0.048 (7)*	
H11B	0.1432 (18)	0.8233 (16)	0.4995 (14)	0.069 (9)*	
H12B	0.5704 (15)	0.6903 (15)	0.5382 (11)	0.049 (8)*	

### Atomic displacement parameters (Å^2^)

**Table:**

	*U*^11^	*U*^22^	*U*^33^	*U*^12^	*U*^13^	*U*^23^
O1	0.0597 (13)	0.0772 (14)	0.0560 (12)	0.0461 (11)	0.0314 (10)	0.0174 (10)
O2	0.0435 (11)	0.0797 (13)	0.0431 (11)	0.0321 (10)	0.0236 (9)	0.0174 (9)
O3	0.0509 (14)	0.0726 (15)	0.0496 (12)	0.0228 (12)	0.0170 (11)	−0.0068 (11)
O4	0.0552 (14)	0.1098 (18)	0.0406 (13)	0.0169 (13)	0.0156 (11)	−0.0074 (12)
O5	0.0549 (12)	0.0731 (13)	0.0429 (11)	0.0418 (11)	0.0143 (9)	0.0183 (9)
O6	0.0514 (11)	0.0722 (13)	0.0305 (10)	0.0289 (10)	0.0060 (9)	0.0096 (9)
O7	0.0554 (14)	0.0656 (14)	0.0470 (12)	0.0218 (12)	0.0214 (10)	0.0197 (10)
O8	0.0537 (13)	0.0957 (16)	0.0469 (13)	0.0176 (11)	0.0235 (11)	0.0183 (11)
O9	0.0572 (12)	0.0552 (12)	0.0468 (11)	0.0332 (10)	0.0013 (9)	0.0096 (9)
O10	0.0417 (10)	0.0694 (12)	0.0323 (10)	0.0268 (9)	0.0144 (8)	0.0087 (9)
O11	0.0595 (13)	0.0595 (13)	0.0523 (12)	0.0251 (11)	0.0351 (10)	0.0145 (10)
O12	0.0525 (13)	0.0704 (14)	0.0423 (12)	0.0164 (10)	0.0197 (10)	−0.0045 (10)
O13	0.0498 (12)	0.0578 (12)	0.0648 (12)	0.0289 (10)	0.0317 (10)	0.0136 (9)
O14	0.0454 (11)	0.0672 (12)	0.0324 (10)	0.0247 (9)	0.0188 (9)	0.0079 (9)
O15	0.0686 (13)	0.0596 (14)	0.0331 (11)	0.0310 (12)	0.0048 (9)	0.0052 (10)
O16	0.0522 (13)	0.0697 (14)	0.0436 (12)	0.0154 (10)	0.0162 (10)	0.0154 (11)
N1	0.0381 (12)	0.0498 (13)	0.0312 (11)	0.0256 (10)	0.0121 (10)	0.0097 (10)
N2	0.0517 (15)	0.0619 (16)	0.0417 (13)	0.0298 (13)	0.0263 (12)	0.0115 (11)
N3	0.0378 (14)	0.0549 (15)	0.0330 (13)	0.0196 (12)	0.0018 (11)	0.0042 (11)
N4	0.0421 (13)	0.0528 (13)	0.0291 (12)	0.0239 (11)	0.0143 (10)	0.0096 (10)
N5	0.0465 (15)	0.0574 (15)	0.0371 (14)	0.0279 (13)	0.0110 (11)	0.0079 (11)
N6	0.0369 (14)	0.0566 (15)	0.0551 (15)	0.0204 (12)	0.0214 (12)	0.0087 (12)
N7	0.0344 (12)	0.0513 (13)	0.0364 (12)	0.0251 (11)	0.0092 (10)	0.0041 (10)
N8	0.0536 (15)	0.0486 (14)	0.0276 (12)	0.0234 (12)	0.0103 (11)	0.0120 (10)
N9	0.0385 (14)	0.0625 (16)	0.0504 (14)	0.0248 (12)	0.0244 (12)	0.0141 (12)
N10	0.0371 (12)	0.0494 (13)	0.0363 (12)	0.0216 (10)	0.0152 (10)	0.0124 (10)
N11	0.0489 (14)	0.0488 (14)	0.0419 (13)	0.0211 (12)	0.0261 (11)	0.0068 (11)
N12	0.0337 (14)	0.0584 (15)	0.0400 (14)	0.0212 (12)	0.0029 (11)	0.0089 (12)
C1	0.0368 (15)	0.0461 (16)	0.0333 (14)	0.0188 (13)	0.0135 (12)	−0.0013 (12)
C2	0.0347 (14)	0.0404 (15)	0.0267 (13)	0.0142 (12)	0.0102 (11)	−0.0013 (11)
C3	0.0296 (14)	0.0403 (15)	0.0292 (13)	0.0129 (11)	0.0076 (11)	0.0005 (11)
C4	0.0318 (15)	0.0488 (16)	0.0336 (14)	0.0169 (12)	0.0104 (12)	−0.0023 (12)
C5	0.0294 (14)	0.0396 (14)	0.0309 (14)	0.0120 (11)	0.0114 (11)	0.0038 (11)
C6	0.0389 (15)	0.0461 (16)	0.0373 (15)	0.0199 (13)	0.0117 (12)	0.0010 (12)
C7	0.0350 (15)	0.0424 (15)	0.0392 (15)	0.0156 (12)	0.0160 (12)	0.0084 (12)
C8	0.0268 (13)	0.0351 (14)	0.0345 (14)	0.0072 (11)	0.0140 (11)	0.0043 (11)
C9	0.0313 (14)	0.0473 (16)	0.0436 (16)	0.0068 (13)	0.0130 (12)	0.0011 (13)
C10	0.0510 (18)	0.0426 (17)	0.0570 (18)	0.0001 (14)	0.0274 (15)	−0.0108 (14)
C11	0.0557 (19)	0.0393 (16)	0.080 (2)	0.0192 (15)	0.0355 (17)	0.0076 (15)
C12	0.0482 (17)	0.0472 (17)	0.0523 (17)	0.0202 (14)	0.0206 (14)	0.0131 (14)
C13	0.0509 (17)	0.0503 (17)	0.0401 (15)	0.0235 (14)	0.0172 (13)	0.0099 (13)
C14	0.061 (2)	0.078 (2)	0.0418 (16)	0.0408 (17)	0.0166 (15)	0.0090 (15)
C15	0.0472 (16)	0.0549 (17)	0.0383 (15)	0.0188 (14)	0.0189 (13)	0.0102 (13)
C16	0.0465 (18)	0.089 (2)	0.0452 (18)	0.0192 (16)	0.0143 (15)	0.0030 (16)
C17	0.0362 (15)	0.0462 (16)	0.0384 (15)	0.0153 (13)	0.0171 (12)	0.0137 (12)
C18	0.0337 (14)	0.0393 (15)	0.0313 (14)	0.0098 (12)	0.0142 (12)	0.0084 (11)
C19	0.0303 (14)	0.0379 (14)	0.0332 (14)	0.0133 (11)	0.0121 (11)	0.0105 (11)
C20	0.0364 (15)	0.0450 (16)	0.0352 (15)	0.0153 (13)	0.0137 (12)	0.0126 (12)
C21	0.0310 (14)	0.0376 (15)	0.0333 (14)	0.0112 (12)	0.0094 (11)	0.0080 (11)
C22	0.0371 (15)	0.0434 (16)	0.0455 (16)	0.0157 (13)	0.0140 (13)	0.0140 (12)
C23	0.0365 (15)	0.0380 (15)	0.0409 (15)	0.0122 (13)	0.0062 (12)	0.0055 (12)
C24	0.0292 (14)	0.0386 (15)	0.0330 (14)	0.0078 (12)	0.0046 (11)	0.0039 (11)
C25	0.0430 (16)	0.0444 (16)	0.0381 (15)	0.0013 (13)	0.0086 (13)	0.0057 (13)
C26	0.067 (2)	0.0431 (18)	0.0493 (18)	0.0033 (16)	0.0085 (16)	0.0145 (14)
C27	0.072 (2)	0.0398 (18)	0.062 (2)	0.0194 (17)	−0.0010 (17)	0.0062 (15)
C28	0.0518 (18)	0.0496 (18)	0.0556 (19)	0.0239 (15)	0.0059 (15)	−0.0022 (15)
C29	0.0619 (19)	0.0483 (17)	0.0476 (16)	0.0227 (15)	0.0269 (15)	0.0106 (13)
C30	0.066 (2)	0.070 (2)	0.0490 (17)	0.0431 (17)	0.0280 (15)	0.0202 (15)
C31	0.0412 (16)	0.0554 (17)	0.0350 (15)	0.0133 (14)	0.0118 (12)	0.0014 (13)
C32	0.0429 (17)	0.080 (2)	0.0473 (18)	0.0161 (16)	0.0178 (14)	0.0060 (16)
C33	0.0344 (15)	0.0390 (15)	0.0377 (15)	0.0081 (12)	0.0049 (12)	0.0013 (12)
C34	0.0342 (14)	0.0360 (14)	0.0313 (14)	0.0108 (12)	0.0111 (11)	0.0060 (11)
C35	0.0289 (14)	0.0366 (14)	0.0305 (13)	0.0112 (11)	0.0121 (11)	0.0058 (11)
C36	0.0267 (14)	0.0463 (16)	0.0330 (15)	0.0107 (12)	0.0083 (12)	0.0070 (12)
C37	0.0285 (14)	0.0400 (15)	0.0310 (13)	0.0138 (11)	0.0107 (11)	0.0058 (11)
C38	0.0371 (15)	0.0502 (16)	0.0395 (15)	0.0203 (13)	0.0179 (12)	0.0174 (12)
C39	0.0364 (15)	0.0448 (16)	0.0360 (15)	0.0171 (13)	0.0064 (12)	0.0021 (12)
C40	0.0273 (14)	0.0362 (14)	0.0284 (13)	0.0067 (11)	0.0063 (11)	0.0007 (11)
C41	0.0389 (15)	0.0431 (16)	0.0373 (15)	0.0034 (13)	0.0105 (12)	0.0032 (12)
C42	0.063 (2)	0.0462 (17)	0.0385 (16)	−0.0031 (15)	0.0109 (14)	0.0114 (13)
C43	0.068 (2)	0.0400 (17)	0.0516 (19)	0.0206 (16)	0.0008 (16)	0.0070 (14)
C44	0.0538 (18)	0.0551 (19)	0.0487 (18)	0.0284 (15)	0.0104 (15)	0.0039 (15)
C45	0.0318 (15)	0.0484 (16)	0.0490 (16)	0.0172 (13)	0.0121 (12)	0.0018 (13)
C46	0.0417 (16)	0.0485 (17)	0.0480 (16)	0.0142 (13)	0.0172 (13)	0.0031 (13)
C47	0.0443 (16)	0.0616 (18)	0.0315 (14)	0.0211 (14)	0.0150 (12)	0.0036 (13)
C48	0.0599 (19)	0.0575 (18)	0.0363 (15)	0.0292 (16)	0.0164 (14)	0.0038 (13)
C49	0.0338 (15)	0.0360 (15)	0.0478 (16)	0.0100 (12)	0.0165 (13)	0.0035 (12)
C50	0.0335 (14)	0.0332 (14)	0.0332 (14)	0.0078 (11)	0.0121 (12)	0.0004 (11)
C51	0.0291 (13)	0.0354 (14)	0.0292 (13)	0.0095 (11)	0.0086 (11)	0.0003 (11)
C52	0.0314 (14)	0.0402 (15)	0.0341 (15)	0.0122 (12)	0.0084 (12)	0.0019 (12)
C53	0.0304 (14)	0.0352 (14)	0.0305 (13)	0.0116 (11)	0.0107 (11)	0.0038 (11)
C54	0.0364 (15)	0.0463 (16)	0.0380 (15)	0.0150 (13)	0.0112 (12)	−0.0022 (12)
C55	0.0371 (15)	0.0404 (15)	0.0441 (16)	0.0129 (13)	0.0206 (13)	0.0069 (12)
C56	0.0293 (14)	0.0370 (14)	0.0384 (14)	0.0081 (11)	0.0166 (12)	0.0052 (12)
C57	0.0404 (16)	0.0433 (16)	0.0431 (16)	0.0045 (13)	0.0191 (13)	−0.0031 (13)
C58	0.0554 (19)	0.0402 (16)	0.0628 (19)	−0.0009 (14)	0.0311 (16)	−0.0113 (14)
C59	0.071 (2)	0.0365 (17)	0.092 (2)	0.0117 (16)	0.0566 (19)	0.0039 (16)
C60	0.0516 (18)	0.0491 (18)	0.075 (2)	0.0267 (15)	0.0353 (16)	0.0211 (15)
C61	0.0410 (16)	0.0461 (16)	0.0416 (15)	0.0183 (13)	0.0136 (13)	0.0130 (12)
C62	0.0467 (17)	0.0471 (17)	0.0418 (16)	0.0155 (14)	0.0109 (13)	0.0019 (13)
C63	0.0451 (16)	0.0630 (19)	0.0337 (15)	0.0216 (14)	0.0180 (13)	0.0122 (13)
C64	0.0591 (19)	0.0501 (18)	0.0486 (17)	0.0232 (15)	0.0261 (15)	0.0142 (14)

### Geometric parameters (Å, °)

**Table:**

O1—C1	1.212 (2)	C15—H15B	0.9700
O2—C4	1.215 (3)	C16—H16A	0.9700
O3—C14	1.426 (3)	C16—H16B	0.9700
O3—H3	0.84 (3)	C17—C18	1.500 (3)
O4—C16	1.412 (3)	C18—C19	1.358 (3)
O4—H4	0.822 (17)	C19—C20	1.456 (3)
O5—C17	1.214 (2)	C19—C21	1.471 (3)
O6—C20	1.211 (2)	C21—C22	1.365 (3)
O7—C30	1.417 (3)	C21—C24	1.438 (3)
O7—H7	0.864 (17)	C22—H22	0.9300
O8—C32	1.427 (3)	C23—C28	1.392 (3)
O8—H8A	0.846 (18)	C23—C24	1.409 (3)
O9—C33	1.203 (2)	C24—C25	1.395 (3)
O10—C36	1.229 (2)	C25—C26	1.377 (3)
O11—C46	1.416 (3)	C25—H25	0.9300
O11—H11A	0.866 (18)	C26—C27	1.399 (3)
O12—C48	1.423 (3)	C26—H26	0.9300
O12—H12A	0.836 (16)	C27—C28	1.358 (4)
O13—C49	1.207 (2)	C27—H27	0.9300
O14—C52	1.222 (2)	C28—H28	0.9300
O15—C62	1.410 (3)	C29—C30	1.506 (3)
O15—H15	0.838 (16)	C29—H29A	0.9700
O16—C64	1.420 (3)	C29—H29B	0.9700
O16—H16	0.831 (17)	C30—H30A	0.9700
N1—C1	1.362 (3)	C30—H30B	0.9700
N1—C4	1.413 (2)	C31—C32	1.490 (3)
N1—C13	1.452 (3)	C31—H31A	0.9700
N2—C2	1.326 (3)	C31—H31B	0.9700
N2—C15	1.463 (3)	C32—H32A	0.9700
N2—H2	0.86 (3)	C32—H32B	0.9700
N3—C6	1.365 (3)	C33—C34	1.502 (3)
N3—C7	1.368 (3)	C34—C35	1.368 (3)
N3—H3A	0.87 (2)	C35—C36	1.444 (3)
N4—C17	1.354 (3)	C35—C37	1.464 (3)
N4—C20	1.418 (3)	C37—C38	1.366 (3)
N4—C29	1.454 (3)	C37—C40	1.432 (3)
N5—C18	1.344 (3)	C38—H38	0.9300
N5—C31	1.450 (3)	C39—C44	1.393 (3)
N5—H5	0.829 (15)	C39—C40	1.409 (3)
N6—C22	1.367 (3)	C40—C41	1.399 (3)
N6—C23	1.368 (3)	C41—C42	1.384 (3)
N6—H6A	0.835 (15)	C41—H41	0.9300
N7—C33	1.384 (3)	C42—C43	1.399 (3)
N7—C36	1.400 (2)	C42—H42	0.9300
N7—C45	1.453 (2)	C43—C44	1.358 (4)
N8—C34	1.334 (3)	C43—H43	0.9300
N8—C47	1.449 (3)	C44—H44	0.9300
N8—H8B	0.886 (15)	C45—C46	1.509 (3)
N9—C38	1.361 (3)	C45—H45A	0.9700
N9—C39	1.364 (3)	C45—H45B	0.9700
N9—H9A	0.890 (16)	C46—H46A	0.9700
N10—C49	1.376 (3)	C46—H46B	0.9700
N10—C52	1.403 (2)	C47—C48	1.507 (3)
N10—C61	1.457 (3)	C47—H47A	0.9700
N11—C50	1.339 (3)	C47—H47B	0.9700
N11—C63	1.457 (3)	C48—H48A	0.9700
N11—H11B	0.878 (15)	C48—H48B	0.9700
N12—C54	1.368 (3)	C49—C50	1.513 (3)
N12—C55	1.369 (3)	C50—C51	1.370 (3)
N12—H12B	0.848 (15)	C51—C52	1.459 (3)
C1—C2	1.511 (3)	C51—C53	1.463 (3)
C2—C3	1.364 (3)	C53—C54	1.372 (3)
C3—C4	1.455 (3)	C53—C56	1.441 (3)
C3—C5	1.468 (3)	C54—H54	0.9300
C5—C6	1.365 (3)	C55—C60	1.391 (3)
C5—C8	1.436 (3)	C55—C56	1.414 (3)
C6—H6	0.9300	C56—C57	1.400 (3)
C7—C12	1.388 (3)	C57—C58	1.367 (3)
C7—C8	1.404 (3)	C57—H57	0.9300
C8—C9	1.396 (3)	C58—C59	1.392 (3)
C9—C10	1.378 (3)	C58—H58	0.9300
C9—H9	0.9300	C59—C60	1.378 (3)
C10—C11	1.395 (3)	C59—H59	0.9300
C10—H10	0.9300	C60—H60	0.9300
C11—C12	1.358 (3)	C61—C62	1.515 (3)
C11—H11	0.9300	C61—H61A	0.9700
C12—H12	0.9300	C61—H61B	0.9700
C13—C14	1.500 (3)	C62—H62A	0.9700
C13—H13A	0.9700	C62—H62B	0.9700
C13—H13B	0.9700	C63—C64	1.505 (3)
C14—H14A	0.9700	C63—H63A	0.9700
C14—H14B	0.9700	C63—H63B	0.9700
C15—C16	1.495 (3)	C64—H64A	0.9700
C15—H15A	0.9700	C64—H64B	0.9700
			
C14—O3—H3	108 (2)	C30—C29—H29A	109.0
C16—O4—H4	111 (2)	N4—C29—H29B	109.0
C30—O7—H7	111 (2)	C30—C29—H29B	109.0
C32—O8—H8A	111 (2)	H29A—C29—H29B	107.8
C46—O11—H11A	107 (2)	O7—C30—C29	108.9 (2)
C48—O12—H12A	104 (2)	O7—C30—H30A	109.9
C62—O15—H15	120 (2)	C29—C30—H30A	109.9
C64—O16—H16	100 (2)	O7—C30—H30B	109.9
C1—N1—C4	109.12 (19)	C29—C30—H30B	109.9
C1—N1—C13	125.62 (19)	H30A—C30—H30B	108.3
C4—N1—C13	125.0 (2)	N5—C31—C32	113.1 (2)
C2—N2—C15	123.8 (2)	N5—C31—H31A	109.0
C2—N2—H2	121.5 (19)	C32—C31—H31A	109.0
C15—N2—H2	114.3 (19)	N5—C31—H31B	109.0
C6—N3—C7	108.72 (19)	C32—C31—H31B	109.0
C6—N3—H3A	126.6 (15)	H31A—C31—H31B	107.8
C7—N3—H3A	124.6 (15)	O8—C32—C31	109.1 (2)
C17—N4—C20	109.35 (18)	O8—C32—H32A	109.9
C17—N4—C29	124.79 (19)	C31—C32—H32A	109.9
C20—N4—C29	125.17 (19)	O8—C32—H32B	109.9
C18—N5—C31	124.7 (2)	C31—C32—H32B	109.9
C18—N5—H5	113.3 (16)	H32A—C32—H32B	108.3
C31—N5—H5	121.6 (16)	O9—C33—N7	126.2 (2)
C22—N6—C23	109.1 (2)	O9—C33—C34	128.0 (2)
C22—N6—H6A	132.4 (18)	N7—C33—C34	105.74 (19)
C23—N6—H6A	118.1 (17)	N8—C34—C35	134.2 (2)
C33—N7—C36	109.16 (18)	N8—C34—C33	117.10 (19)
C33—N7—C45	125.60 (18)	C35—C34—C33	108.75 (19)
C36—N7—C45	124.32 (19)	C34—C35—C36	106.81 (19)
C34—N8—C47	125.5 (2)	C34—C35—C37	130.6 (2)
C34—N8—H8B	116.7 (14)	C36—C35—C37	122.42 (19)
C47—N8—H8B	114.6 (14)	O10—C36—N7	121.5 (2)
C38—N9—C39	108.7 (2)	O10—C36—C35	129.2 (2)
C38—N9—H9A	124.7 (18)	N7—C36—C35	109.28 (19)
C39—N9—H9A	126.6 (18)	C38—C37—C40	106.50 (19)
C49—N10—C52	109.52 (19)	C38—C37—C35	124.5 (2)
C49—N10—C61	124.06 (19)	C40—C37—C35	129.0 (2)
C52—N10—C61	125.21 (19)	N9—C38—C37	110.3 (2)
C50—N11—C63	125.1 (2)	N9—C38—H38	124.9
C50—N11—H11B	118.9 (16)	C37—C38—H38	124.9
C63—N11—H11B	113.3 (17)	N9—C39—C44	129.3 (2)
C54—N12—C55	109.22 (19)	N9—C39—C40	108.3 (2)
C54—N12—H12B	124.6 (15)	C44—C39—C40	122.4 (3)
C55—N12—H12B	125.8 (15)	C41—C40—C39	118.8 (2)
O1—C1—N1	126.0 (2)	C41—C40—C37	135.0 (2)
O1—C1—C2	127.2 (2)	C39—C40—C37	106.2 (2)
N1—C1—C2	106.81 (19)	C42—C41—C40	118.6 (2)
N2—C2—C3	134.6 (2)	C42—C41—H41	120.7
N2—C2—C1	117.1 (2)	C40—C41—H41	120.7
C3—C2—C1	108.3 (2)	C41—C42—C43	120.8 (3)
C2—C3—C4	106.90 (19)	C41—C42—H42	119.6
C2—C3—C5	130.7 (2)	C43—C42—H42	119.6
C4—C3—C5	122.2 (2)	C44—C43—C42	122.1 (2)
O2—C4—N1	121.1 (2)	C44—C43—H43	118.9
O2—C4—C3	130.0 (2)	C42—C43—H43	118.9
N1—C4—C3	108.9 (2)	C43—C44—C39	117.2 (3)
C6—C5—C8	105.93 (19)	C43—C44—H44	121.4
C6—C5—C3	126.5 (2)	C39—C44—H44	121.4
C8—C5—C3	127.6 (2)	N7—C45—C46	111.06 (19)
C5—C6—N3	110.5 (2)	N7—C45—H45A	109.4
C5—C6—H6	124.7	C46—C45—H45A	109.4
N3—C6—H6	124.7	N7—C45—H45B	109.4
N3—C7—C12	129.6 (2)	C46—C45—H45B	109.4
N3—C7—C8	107.84 (19)	H45A—C45—H45B	108.0
C12—C7—C8	122.6 (2)	O11—C46—C45	111.87 (19)
C9—C8—C7	118.9 (2)	O11—C46—H46A	109.2
C9—C8—C5	134.2 (2)	C45—C46—H46A	109.2
C7—C8—C5	106.97 (19)	O11—C46—H46B	109.2
C10—C9—C8	118.4 (2)	C45—C46—H46B	109.2
C10—C9—H9	120.8	H46A—C46—H46B	107.9
C8—C9—H9	120.8	N8—C47—C48	112.0 (2)
C9—C10—C11	121.2 (2)	N8—C47—H47A	109.2
C9—C10—H10	119.4	C48—C47—H47A	109.2
C11—C10—H10	119.4	N8—C47—H47B	109.2
C12—C11—C10	121.8 (2)	C48—C47—H47B	109.2
C12—C11—H11	119.1	H47A—C47—H47B	107.9
C10—C11—H11	119.1	O12—C48—C47	112.37 (19)
C11—C12—C7	117.2 (2)	O12—C48—H48A	109.1
C11—C12—H12	121.4	C47—C48—H48A	109.1
C7—C12—H12	121.4	O12—C48—H48B	109.1
N1—C13—C14	112.7 (2)	C47—C48—H48B	109.1
N1—C13—H13A	109.1	H48A—C48—H48B	107.9
C14—C13—H13A	109.1	O13—C49—N10	127.0 (2)
N1—C13—H13B	109.1	O13—C49—C50	126.6 (2)
C14—C13—H13B	109.1	N10—C49—C50	106.34 (19)
H13A—C13—H13B	107.8	N11—C50—C51	135.2 (2)
O3—C14—C13	109.3 (2)	N11—C50—C49	116.7 (2)
O3—C14—H14A	109.8	C51—C50—C49	108.1 (2)
C13—C14—H14A	109.8	C50—C51—C52	107.10 (19)
O3—C14—H14B	109.8	C50—C51—C53	129.9 (2)
C13—C14—H14B	109.8	C52—C51—C53	122.75 (19)
H14A—C14—H14B	108.3	O14—C52—N10	122.5 (2)
N2—C15—C16	114.1 (2)	O14—C52—C51	128.7 (2)
N2—C15—H15A	108.7	N10—C52—C51	108.79 (19)
C16—C15—H15A	108.7	C54—C53—C56	106.44 (19)
N2—C15—H15B	108.7	C54—C53—C51	125.21 (19)
C16—C15—H15B	108.7	C56—C53—C51	128.4 (2)
H15A—C15—H15B	107.6	N12—C54—C53	110.0 (2)
O4—C16—C15	108.7 (2)	N12—C54—H54	125.0
O4—C16—H16A	110.0	C53—C54—H54	125.0
C15—C16—H16A	110.0	N12—C55—C60	130.0 (2)
O4—C16—H16B	110.0	N12—C55—C56	107.84 (19)
C15—C16—H16B	110.0	C60—C55—C56	122.1 (2)
H16A—C16—H16B	108.3	C57—C56—C55	118.5 (2)
O5—C17—N4	126.8 (2)	C57—C56—C53	135.0 (2)
O5—C17—C18	126.4 (2)	C55—C56—C53	106.50 (19)
N4—C17—C18	106.73 (19)	C58—C57—C56	119.2 (2)
N5—C18—C19	133.9 (2)	C58—C57—H57	120.4
N5—C18—C17	117.3 (2)	C56—C57—H57	120.4
C19—C18—C17	108.8 (2)	C57—C58—C59	121.4 (2)
C18—C19—C20	106.70 (19)	C57—C58—H58	119.3
C18—C19—C21	131.2 (2)	C59—C58—H58	119.3
C20—C19—C21	121.95 (19)	C60—C59—C58	121.5 (2)
O6—C20—N4	122.2 (2)	C60—C59—H59	119.3
O6—C20—C19	129.5 (2)	C58—C59—H59	119.3
N4—C20—C19	108.30 (19)	C59—C60—C55	117.3 (2)
C22—C21—C24	106.0 (2)	C59—C60—H60	121.4
C22—C21—C19	126.2 (2)	C55—C60—H60	121.4
C24—C21—C19	127.8 (2)	N10—C61—C62	111.7 (2)
C21—C22—N6	110.4 (2)	N10—C61—H61A	109.3
C21—C22—H22	124.8	C62—C61—H61A	109.3
N6—C22—H22	124.8	N10—C61—H61B	109.3
N6—C23—C28	130.4 (3)	C62—C61—H61B	109.3
N6—C23—C24	107.4 (2)	H61A—C61—H61B	107.9
C28—C23—C24	122.2 (3)	O15—C62—C61	111.0 (2)
C25—C24—C23	118.9 (2)	O15—C62—H62A	109.4
C25—C24—C21	134.0 (2)	C61—C62—H62A	109.4
C23—C24—C21	107.1 (2)	O15—C62—H62B	109.4
C26—C25—C24	118.9 (3)	C61—C62—H62B	109.4
C26—C25—H25	120.6	H62A—C62—H62B	108.0
C24—C25—H25	120.6	N11—C63—C64	111.08 (19)
C25—C26—C27	120.6 (3)	N11—C63—H63A	109.4
C25—C26—H26	119.7	C64—C63—H63A	109.4
C27—C26—H26	119.7	N11—C63—H63B	109.4
C28—C27—C26	122.2 (3)	C64—C63—H63B	109.4
C28—C27—H27	118.9	H63A—C63—H63B	108.0
C26—C27—H27	118.9	O16—C64—C63	112.60 (19)
C27—C28—C23	117.2 (3)	O16—C64—H64A	109.1
C27—C28—H28	121.4	C63—C64—H64A	109.1
C23—C28—H28	121.4	O16—C64—H64B	109.1
N4—C29—C30	112.8 (2)	C63—C64—H64B	109.1
N4—C29—H29A	109.0	H64A—C64—H64B	107.8
			
C4—N1—C1—O1	179.4 (2)	C36—N7—C33—O9	−174.9 (2)
C13—N1—C1—O1	4.7 (4)	C45—N7—C33—O9	−5.6 (4)
C4—N1—C1—C2	−1.2 (2)	C36—N7—C33—C34	5.2 (2)
C13—N1—C1—C2	−175.9 (2)	C45—N7—C33—C34	174.5 (2)
C15—N2—C2—C3	−9.9 (4)	C47—N8—C34—C35	19.5 (4)
C15—N2—C2—C1	168.2 (2)	C47—N8—C34—C33	−161.0 (2)
O1—C1—C2—N2	2.2 (4)	O9—C33—C34—N8	−4.4 (4)
N1—C1—C2—N2	−177.1 (2)	N7—C33—C34—N8	175.5 (2)
O1—C1—C2—C3	−179.2 (2)	O9—C33—C34—C35	175.1 (3)
N1—C1—C2—C3	1.4 (2)	N7—C33—C34—C35	−4.9 (3)
N2—C2—C3—C4	177.2 (3)	N8—C34—C35—C36	−177.8 (3)
C1—C2—C3—C4	−1.0 (2)	C33—C34—C35—C36	2.7 (3)
N2—C2—C3—C5	−7.8 (4)	N8—C34—C35—C37	6.9 (5)
C1—C2—C3—C5	173.9 (2)	C33—C34—C35—C37	−172.6 (2)
C1—N1—C4—O2	−179.2 (2)	C33—N7—C36—O10	175.0 (2)
C13—N1—C4—O2	−4.4 (3)	C45—N7—C36—O10	5.5 (4)
C1—N1—C4—C3	0.6 (2)	C33—N7—C36—C35	−3.7 (3)
C13—N1—C4—C3	175.3 (2)	C45—N7—C36—C35	−173.2 (2)
C2—C3—C4—O2	−179.9 (2)	C34—C35—C36—O10	−178.2 (2)
C5—C3—C4—O2	4.6 (4)	C37—C35—C36—O10	−2.4 (4)
C2—C3—C4—N1	0.3 (2)	C34—C35—C36—N7	0.4 (3)
C5—C3—C4—N1	−175.19 (19)	C37—C35—C36—N7	176.3 (2)
C2—C3—C5—C6	−58.8 (4)	C34—C35—C37—C38	45.4 (4)
C4—C3—C5—C6	115.5 (3)	C36—C35—C37—C38	−129.3 (2)
C2—C3—C5—C8	120.0 (3)	C34—C35—C37—C40	−134.2 (3)
C4—C3—C5—C8	−65.7 (3)	C36—C35—C37—C40	51.1 (3)
C8—C5—C6—N3	−1.8 (3)	C39—N9—C38—C37	−1.4 (3)
C3—C5—C6—N3	177.2 (2)	C40—C37—C38—N9	2.2 (3)
C7—N3—C6—C5	1.4 (3)	C35—C37—C38—N9	−177.46 (19)
C6—N3—C7—C12	−179.8 (3)	C38—N9—C39—C44	−179.7 (2)
C6—N3—C7—C8	−0.3 (3)	C38—N9—C39—C40	0.0 (3)
N3—C7—C8—C9	179.0 (2)	N9—C39—C40—C41	−178.0 (2)
C12—C7—C8—C9	−1.4 (4)	C44—C39—C40—C41	1.8 (3)
N3—C7—C8—C5	−0.9 (3)	N9—C39—C40—C37	1.3 (2)
C12—C7—C8—C5	178.8 (2)	C44—C39—C40—C37	−179.0 (2)
C6—C5—C8—C9	−178.2 (3)	C38—C37—C40—C41	177.0 (2)
C3—C5—C8—C9	2.8 (4)	C35—C37—C40—C41	−3.4 (4)
C6—C5—C8—C7	1.6 (3)	C38—C37—C40—C39	−2.1 (2)
C3—C5—C8—C7	−177.4 (2)	C35—C37—C40—C39	177.5 (2)
C7—C8—C9—C10	0.4 (3)	C39—C40—C41—C42	0.3 (3)
C5—C8—C9—C10	−179.8 (3)	C37—C40—C41—C42	−178.7 (2)
C8—C9—C10—C11	0.8 (4)	C40—C41—C42—C43	−2.0 (3)
C9—C10—C11—C12	−1.1 (4)	C41—C42—C43—C44	1.7 (4)
C10—C11—C12—C7	0.2 (4)	C42—C43—C44—C39	0.3 (4)
N3—C7—C12—C11	−179.4 (3)	N9—C39—C44—C43	177.6 (2)
C8—C7—C12—C11	1.1 (4)	C40—C39—C44—C43	−2.1 (4)
C1—N1—C13—C14	−83.0 (3)	C33—N7—C45—C46	89.9 (3)
C4—N1—C13—C14	103.1 (3)	C36—N7—C45—C46	−102.2 (2)
N1—C13—C14—O3	−62.8 (3)	N7—C45—C46—O11	166.08 (18)
C2—N2—C15—C16	108.6 (3)	C34—N8—C47—C48	60.6 (3)
N2—C15—C16—O4	70.6 (3)	N8—C47—C48—O12	60.5 (3)
C20—N4—C17—O5	178.5 (2)	C52—N10—C49—O13	175.2 (2)
C29—N4—C17—O5	7.6 (4)	C61—N10—C49—O13	7.2 (4)
C20—N4—C17—C18	−2.5 (3)	C52—N10—C49—C50	−4.3 (2)
C29—N4—C17—C18	−173.3 (2)	C61—N10—C49—C50	−172.23 (19)
C31—N5—C18—C19	−11.6 (4)	C63—N11—C50—C51	−22.0 (4)
C31—N5—C18—C17	167.7 (2)	C63—N11—C50—C49	157.6 (2)
O5—C17—C18—N5	2.7 (4)	O13—C49—C50—N11	4.3 (4)
N4—C17—C18—N5	−176.3 (2)	N10—C49—C50—N11	−176.2 (2)
O5—C17—C18—C19	−177.8 (2)	O13—C49—C50—C51	−176.0 (2)
N4—C17—C18—C19	3.1 (3)	N10—C49—C50—C51	3.5 (2)
N5—C18—C19—C20	176.9 (3)	N11—C50—C51—C52	178.3 (3)
C17—C18—C19—C20	−2.4 (3)	C49—C50—C51—C52	−1.3 (2)
N5—C18—C19—C21	−8.3 (5)	N11—C50—C51—C53	−7.0 (4)
C17—C18—C19—C21	172.4 (2)	C49—C50—C51—C53	173.4 (2)
C17—N4—C20—O6	−177.9 (2)	C49—N10—C52—O14	−175.1 (2)
C29—N4—C20—O6	−7.1 (4)	C61—N10—C52—O14	−7.4 (3)
C17—N4—C20—C19	1.0 (3)	C49—N10—C52—C51	3.6 (3)
C29—N4—C20—C19	171.9 (2)	C61—N10—C52—C51	171.4 (2)
C18—C19—C20—O6	179.8 (3)	C50—C51—C52—O14	177.4 (2)
C21—C19—C20—O6	4.4 (4)	C53—C51—C52—O14	2.2 (4)
C18—C19—C20—N4	1.0 (3)	C50—C51—C52—N10	−1.3 (2)
C21—C19—C20—N4	−174.4 (2)	C53—C51—C52—N10	−176.44 (19)
C18—C19—C21—C22	−57.9 (4)	C50—C51—C53—C54	−43.0 (4)
C20—C19—C21—C22	116.2 (3)	C52—C51—C53—C54	130.9 (3)
C18—C19—C21—C24	122.4 (3)	C50—C51—C53—C56	136.3 (3)
C20—C19—C21—C24	−63.4 (3)	C52—C51—C53—C56	−49.8 (3)
C24—C21—C22—N6	−1.5 (3)	C55—N12—C54—C53	1.3 (3)
C19—C21—C22—N6	178.8 (2)	C56—C53—C54—N12	−1.9 (3)
C23—N6—C22—C21	1.2 (3)	C51—C53—C54—N12	177.6 (2)
C22—N6—C23—C28	−179.7 (2)	C54—N12—C55—C60	−179.2 (3)
C22—N6—C23—C24	−0.4 (3)	C54—N12—C55—C56	−0.2 (3)
N6—C23—C24—C25	180.0 (2)	N12—C55—C56—C57	178.2 (2)
C28—C23—C24—C25	−0.7 (3)	C60—C55—C56—C57	−2.8 (4)
N6—C23—C24—C21	−0.5 (2)	N12—C55—C56—C53	−0.9 (3)
C28—C23—C24—C21	178.8 (2)	C60—C55—C56—C53	178.1 (2)
C22—C21—C24—C25	−179.3 (3)	C54—C53—C56—C57	−177.2 (3)
C19—C21—C24—C25	0.4 (4)	C51—C53—C56—C57	3.4 (4)
C22—C21—C24—C23	1.2 (2)	C54—C53—C56—C55	1.7 (3)
C19—C21—C24—C23	−179.1 (2)	C51—C53—C56—C55	−177.7 (2)
C23—C24—C25—C26	−0.2 (3)	C55—C56—C57—C58	1.3 (4)
C21—C24—C25—C26	−179.6 (2)	C53—C56—C57—C58	−180.0 (3)
C24—C25—C26—C27	1.0 (4)	C56—C57—C58—C59	0.5 (4)
C25—C26—C27—C28	−1.0 (4)	C57—C58—C59—C60	−1.0 (4)
C26—C27—C28—C23	0.1 (4)	C58—C59—C60—C55	−0.4 (4)
N6—C23—C28—C27	179.9 (3)	N12—C55—C60—C59	−178.9 (3)
C24—C23—C28—C27	0.8 (4)	C56—C55—C60—C59	2.3 (4)
C17—N4—C29—C30	−77.1 (3)	C49—N10—C61—C62	−80.2 (3)
C20—N4—C29—C30	113.5 (3)	C52—N10—C61—C62	113.8 (2)
N4—C29—C30—O7	−64.3 (3)	N10—C61—C62—O15	−170.44 (18)
C18—N5—C31—C32	112.1 (3)	C50—N11—C63—C64	−61.8 (3)
N5—C31—C32—O8	65.4 (3)	N11—C63—C64—O16	−60.6 (3)

### Hydrogen-bond geometry (Å, °)

**Table:**

*D*—H···*A*	*D*—H	H···*A*	*D*···*A*	*D*—H···*A*
N2—H2···O12^i^	0.86 (3)	2.09 (3)	2.950 (3)	177 (3)
O3—H3···O8^ii^	0.84 (3)	1.93 (3)	2.762 (3)	172 (3)
O4—H4···O14^iii^	0.82 (2)	1.97 (2)	2.770 (2)	163 (3)
N5—H5···O16^iv^	0.83 (2)	2.22 (2)	3.027 (3)	164 (2)
O7—H7···O4^v^	0.86 (2)	1.89 (2)	2.755 (3)	175 (3)
O15—H15···O1^vi^	0.84 (2)	2.22 (2)	2.893 (2)	137 (3)
O15—H15···O12^vii^	0.84 (2)	2.42 (2)	3.097 (3)	139 (3)
O16—H16···O11^i^	0.83 (2)	1.96 (2)	2.780 (3)	170 (3)
N3—H3A···O6^iii^	0.87 (2)	2.06 (2)	2.840 (2)	149 (2)
N6—H6A···O14	0.84 (2)	2.22 (2)	3.042 (3)	168 (2)
O8—H8A···O10^viii^	0.85 (2)	1.90 (2)	2.746 (3)	173 (3)
N9—H9A···O2^viii^	0.89 (2)	1.95 (2)	2.760 (2)	152 (2)
O11—H11A···O5^ii^	0.87 (2)	2.13 (2)	2.935 (2)	154 (3)
O11—H11A···O16	0.87 (2)	2.51 (3)	3.033 (3)	120 (3)
O12—H12A···O15^ix^	0.84 (2)	1.92 (2)	2.739 (2)	168 (3)
N8—H8B···O7^x^	0.89 (2)	2.09 (2)	2.867 (3)	146 (2)
N11—H11B···O3	0.88 (2)	2.17 (2)	2.945 (3)	147 (2)
N12—H12B···O10^viii^	0.85 (2)	2.10 (2)	2.925 (2)	164 (2)

Symmetry codes: (i) −*x*, −*y*+1, −*z*+1; (ii) *x*−1, *y*, *z*; (iii) *x*, *y*, *z*+1; (iv) *x*+1, *y*, *z*; (v) *x*+1, *y*, *z*−1; (vi) −*x*, −*y*+2, −*z*+1; (vii) *x*, *y*+1, *z*; (viii) −*x*+1, −*y*+1, −*z*+1; (ix) −*x*, −*y*+1, −*z*; (x) −*x*+1, −*y*+1, −*z*.
